# *Chrysophyllum cainito* stem bark extract induces apoptosis in Human hepatocarcinoma HepG2 cells through ROS-mediated mitochondrial pathway

**DOI:** 10.7717/peerj.10168

**Published:** 2020-10-16

**Authors:** Hau V. Doan, Pishyaporn Sritangos, Roongtip Iyara, Nuannoi Chudapongse

**Affiliations:** 1 Department of Pharmacy, School of Medicine and Pharmacy, Tra Vinh University, Tra Vinh, Vietnam; 2 School of Preclinical Sciences, Institute of Science, Suranaree University of Technology, Muang, Nakhon Ratchasima, Thailand

**Keywords:** Chrysophyllum cainito, Apoptosis, Hepatocellular carcinoma, Reactive oxygen species, Cell cycles, HepG2 cell

## Abstract

Hepatocellular carcinoma is the most common type of primary liver cancer in humans. This study aimed to demonstrate anticancer properties of an aqueous extract from *Chrysophyllum cainito* stem bark (CE) and its underlying mechanisms. Our MTT assay results showed that CE significantly reduced human hepatocellular carcinoma (HepG2) cell viability with the IC_50_of 100 µg/mL, while human dermal primary fibroblast (HDFa) cells showed less susceptibility in every concentration tested. Determined by Annexin V staining, the proportion of apoptotic HepG2 cells increased in a dose-dependent fashion after 24 hour-exposure of CE. The results from Western blot analysis confirmed that CE reduced procaspase-3, suggesting apoptosis by activating caspase-3 cleavage. Using the DCFH-DA and DiOC6 fluorescent probes, it was found that CE significantly stimulated the generation of reactive oxygen species (ROS) and reduced mitochondrial membrane potential (Δ*ψ*_m_), respectively. According to cell cycle analysis, CE (100 µg/mL) profoundly increased the percentage of cells in the sub-G1 phase, indicating cell apoptosis. These data suggest that CE induces apoptosis and cell death in human hepatocellular carcinoma via generation of intracellular ROS and disruption of Δ*ψ*m. This is the first demonstration of the anticancer activity with proposed underlying mechanism of CE in liver cancer cells.

## Introduction

Liver cancer is one of the most commonly diagnosed cancers and the leading cause of cancer-related death worldwide (Global Burden of Disease Liver Cancer Collaboration, 2017; Siegel, Miller & Jemal, 2017). The most common form of liver cancer is hepatocellular carcinoma (HCC). The incidences of HCC have increased by 4-fold over the last four decades and is projected to further increase. The development of HCC is associated with a number of risk factors including liver cirrhosis, hepatitis B and C virus, heavy smoking, alcoholic and non-alcoholic liver diseases (Massarweh & El-Serag, 2017). Although resection is the most curative therapy for HCC, chemotherapy remains a primary treatment for most cases (Johnson, 2005). However, more than half of HCC patients who did not undergo resection died from disease progression (Couto, Dvorchik & Carr, 2007). As Asia-Pacific and sub-Saharan Africa regions account for 82% of HCC cases worldwide (Zhu et al., 2016), HCC is considered as a major public health burden within these regions.

Medicinal plants and herbal preparations have been traditionally used to treat diseases. Medicinal plants contain a vast amount of phytochemicals which may exert anticancer effects, therefore, remains as a valuable resource for drug discovery (Shukla & Mehta, 2015).

*Chrysophyllum cainito* L. is an erected tropical fruit tree that belongs to the genus *Chrysophyllum* in Sapotaceae family. *C*. *cainito* is known by many different names, including: star apple, caimito, cainito, milk fruit, golden leaf tree (Das, Nordin & Bhaumik, 2010). In traditional medicine, the leaf, fruit and stem bark of *C*. *cainito* have been used for its antidiabetes (Koffi et al., 2009; Koffi, Konan Édouard & Kouassi, 2009), antioxidant (Luo, Basile & Kennelly, 2002), anti-inflammation, anti-hypertension (Meira et al., 2014) and antibacterial activities (Oranusi, Braide & Umeze, 2015). A polyphenolic fraction from *C*. *cainito* pulp was shown to inhibit the growth of osteosarcoma cells (Li et al., 2015), indicating a potential anticancer effect.

Apoptosis is a programmed cell death triggered by either intrinsic or extrinsic signaling pathways resulting in the removal of damaged cells at the molecular level. Apoptosis resistance is a key hallmark of cancer which enables cancer cells to continuously accumulate mutations while avoiding cell death (Hanahan & Weinberg, 2000). Anticancer agents can mechanistically promote apoptosis through either the death receptor pathway (extrinsic pathway) or the mitochondrial apoptosis pathway (intrinsic pathway) (Fulda, 2015). The mitochondrial apoptosis pathway typically involves the generation of reactive oxygen species (ROS) and the disruption of mitochondrial membrane potential (Δ*ψ*m) leading to the release of cytochrome c (Simon, Haj-Yehia & Levi-Schaffer, 2000).

In the present study, the anticancer effects of the *cainito* stem bark extract (CE) were evaluated using human hepatocarcinoma HepG2 cell cell line and non-cancerous fibroblast cell lines. We showed that CE significantly induced HepG2 cancer cell death in comparison to the non-cancerous fibroblasts. The possible mechanism of HepG2 cell death was investigated using ROS detection assays and measurement of the mitochondrial membrane potential. The current study provides the first evidence that *C*. *cainito* stem bark extract exerts anticancer effect by inducing apoptotic cell death in HepG2 hepatocarcinoma cells.

## Materials & Methods

### Chemicals

Dulbecco’s modified eagle’s medium (DMEM), fetal bovine serum (FBS), 4-(2-hydroxyethyl)piperazine-1-ethanesulfonic acid (HEPES, 1 M), and penicillin-streptomycin (Pen-strep) were purchased from Gibco (New York, USA). 3-(4,5-dimethylthiazol-2-yl)-2,5-diphenyltetrazolium bromide (MTT), 2′,7′-dichlorofluorescin diacetate (DCFH-DA), and 3′3-dihexyloxacarbocyanine (DiOC6(3)) were purchased from Sigma-Aldrich (Missouri, USA). Tali™ apoptosis and Tali™ cell cycle kits were obtained from Invitrogen (Oregon, USA).

### Plant extraction

The stem bark of *C*. *cainito* was collected from Mo Cay Nam district, Ben Tre, Vietnam. The dried stem was blended using a food blender. The aqueous extract of *C*. *cainito* was obtained by maceration on a shaker for 2 hours/cycle. After each cycle ended, the aqueous fraction was removed, and fresh deionized water was added for the next cycle. Four cycles were performed until the dark brown extract appears to fade in color. All collected aqueous extract fractions were combined together, filtered by a cotton gauze, then centrifuged 15 min at 5,000 rpm to remove remaining debris. The filtered extract solution was concentrated using a rotary evaporator, at 40 °C. The volume was reduced to 5% of the initial volume. The concentrated extract was then freeze-dried by a lyophilizer for two days. The product yield was 11% and the obtained product was brown-colored powder. The *C*. *cainito* dried extract (CE) was kept at −20 °C until used.

### Cells and cell culture

Human liver cancer cells (HepG2) and non-cancerous primary human dermal fibroblasts (HDFa) or mouse fibroblast (NIH/3T3) cell lines were obtained from American Type Culture Collection (ATCC, Virginia, USA). Both cell types were grown in DMEM media supplied with 10% FBS, 1% Pen-strep, and 1% HEPES. Cells were maintained in a humidified incubator at 37 °C, 5% CO_2_.

### Cytotoxicity assay

Cell viability was determined using MTT assay (Mosmann, 1983) where HepG2 (4 × 10^4^ cells/well) or fibroblast cells (2 × 10^4^ cells/well) were plated in a 96-well plate and allowed to adhere overnight. Then, cells were exposed to different concentrations of CE (0 –800 µg/ml) for 24 h. Post-24 h of treatment, the media were aspirated and cells were washed with phosphate buffer saline (PBS, pH 7.4). MTT reagent (100 µL of 0.5 mg/mL MTT) was added to each well then the plate was incubated in a humidified incubator maintained at 37 °C, 5% CO_2_. After 3 h of incubation, MTT was removed from all wells. DMSO (100 µL) was added then further incubated for 10 min to dissolve purple formazan product. Cell viability was measured using spectrophotometry at 570 nm absorbance. Cell viability of treatment conditions was presented as the percentage of viable cells in comparison to the control.

### Apoptosis analysis

Apoptosis was determined using a Tali™ apoptosis kit. The Tali™ apoptosis kit contains fluorophore-labelled Annexin V and propidium iodide conventionally used to assess apoptotic cell death. Annexin V is used to assess apoptotic phosphatidylserine flips, indicative of apoptosis. Propidium iodide is used to assess the loss of membrane integrity which occurs during cell death (Demchenko, 2013; Life Technology, 2013; Zhou, Cui & Urban, 2011).

HepG2 cells were seeded at 5 × 10^5^ cells/ml/well into a 12-well plate. Seeded cells were treated with different concentrations of CE (0, 50, 70, and 100 µg/ml) for 24 h. Cells were harvested using 0.25% trypsin-EDTA, washed with PBS, then stained with the Tali™ apoptosis reagent according to the manufacturer’s protocol. Apoptotic cells stained by Annexin V (green fluorescence) and dead cells stained by propidium iodide (red fluorescence). Stained cells were imaged, counted, and analyzed using a Tali^®^ image-based cytometer (Invitrogen, USA). Data are presented as the percentage of dead and apoptotic cells in comparison to control.

### Western immunoblotting

Cells were treated with either 0 or 100 µg/ml CE for 24 h then harvested using Triton-X lysis buffer (150 mM NaCl, 1.0% Triton-X, 50 mM Tris pH 8.0, 1 mM PMSF). Lysate protein concentrations were measured using Bradford assay (Bio-rad, USA). Equal amounts of protein (10 or 20 µg) were resolved using 12% SDS-PAGE then transferred onto PVDF membranes (Bio-rad, USA). The membranes were blocked with 5% skim milk in Tris buffer saline (20 mM Tris, 150 mM NaCl, 0.1% Tween-20; TBST) for 1 h, at room temperature. The membranes were then incubated overnight with a primary antibody, at 4 °C. After removing the primary antibody and washing with TBST, the membranes were incubated with horseradish peroxidase-conjugated secondary antibody for 2 h at room temperature (Ngernsoungnern & Ngernsoungnern, 2016). Immunoblotting was performed using caspase 3 (1:1000) or *β*-actin (1:2000) primary antibodies (Cell Signaling Technology, USA) and goat-anti-rabbit secondary antibody (Invitrogen, USA) diluted in 1% bovine serum albumin TBST. Protein bands were visualized using DAB kit according to the manufacturer’s instructions (Vector Laboratories, USA). The NIH/3T3 fibroblast cell line was used as a control.

### Cell morphology observation

HepG2 cells were prepared as described in the apoptosis analysis assay. After 24 h of incubation with CE, the morphological changes of HepG2 cells were viewed under an inverted phase contrast microscope (Olympus IX51, Tokyo, Japan).

### Reactive oxygen species (ROS) determination

The intracellular ROS production was determined using DCFH-DA following the method described previously (Halliwell & Whiteman, 2004) with the following modifications. HepG2 cells were seeded at 4 × 10^4^ cells/well into black-wall clear-bottom 96-well plates. Cells were allowed to adhere overnight then treated with CE (0, 50, 75, and 100 µg/ml) for 12 h. Afterwards, cells were washed with PBS twice and incubated with 50 µM DCFH-DA for 45 min, in darkness, at 37 °C. The cells were washed three times with PBS before the fluorescence intensity was determined using a fluorescence microplate reader at Excitation/Emission of 485/530 nm. The measured fluorescence intensity was compared against the control (CE 0 µg/ml), yielding the relative CE-induced ROS generation.

Intracellular ROS was also monitored by fluorescence microscopy. Then HepG2 cells were cultured on glass coverslips and stained by DCFH-DA following the above description. Intracellular fluorescence was detected by a Nikon phase contrast fluorescence microscope (Eclipse 80i, Japan).

### Determination of mitochondrial membrane potential (Δ*ψ*m)

The effect of CE on the level of the Δ*ψ*m of HepG2 cells was measured using DiOC6 probe (Yu et al., 2016). HepG2 cells were seeded at 4 × 10^4^ cells/well in a black-wall clear-bottom 96-well plate. Cells were incubated overnight then treated with 100 µg/ml CE. This concentration was chosen based on its effect on ROS generation. After 12 h of CE treatment, cells were washed with PBS and incubated with DMEM containing 40 nM DiOC6 for 30 min, at 37 °C. Cells were further washed with PBS prior to the fluorescence intensity measurement using a fluorescence plate reader at Excitation/Emission of 488/525 nm. The positive control condition was treated with 50 µM CCCP, 1 h, to demonstrate depolarization of Δ *ψ*m.

### Cell cycle analysis

Cell cycle distribution of HepG2 cells was analyzed using a Tali™ cell cycle kit according to the manufacturer’s instruction. The Tali cell cycle reagent contains propidium iodide, a dye conventionally used to stain DNA for cell cycle monitoring.

HepG2 cells were cultured in FBS-free media for 24 h to deprive cells of growth factors, subsequently enabling the cells to synchronize at the G0/G1 phase (Jia, Han & Chen, 2005). HepG2 cells were then seeded at 5 × 10^5^ cells/ml/well into 12-well plate prior to treatment with different concentrations of CE (0, 50, 70, and 100 µg/ml) for 24 h. Then, cells were harvested using 0.25% trypsin-EDTA and washed with PBS. Cells were fixed with ice-cold 70% ethanol overnight, at −20 °C. Cells were stained with Tali™ cell cycle reagent for 30 min. Using the Tali^®^ image-based cytometer (Invitrogen, USA), the proportion of cells at different cell cycle phases were counted and quantified based on the red fluorescence intensity. Gate parameter was 3–28 µm. The minimum number of the events measured was 1,637 cells.

### Statistical analysis

All experiments were done in triplicate per treatment condition. All experiments were independently repeated at least three times. The data presented in this study were tested for normality and were normally distributed. Statistical comparisons between groups were calculated using either One-way or Two-way ANOVA, followed by Student-Newman-Keuls (SNK) post-hoc analysis. All data are expressed as mean ± SEM. A value of *p* <  0.05 was considered statistically significant.

## Results

### Cytotoxic effect of CE on HepG2 and HDFa cells

**Figure 1:**
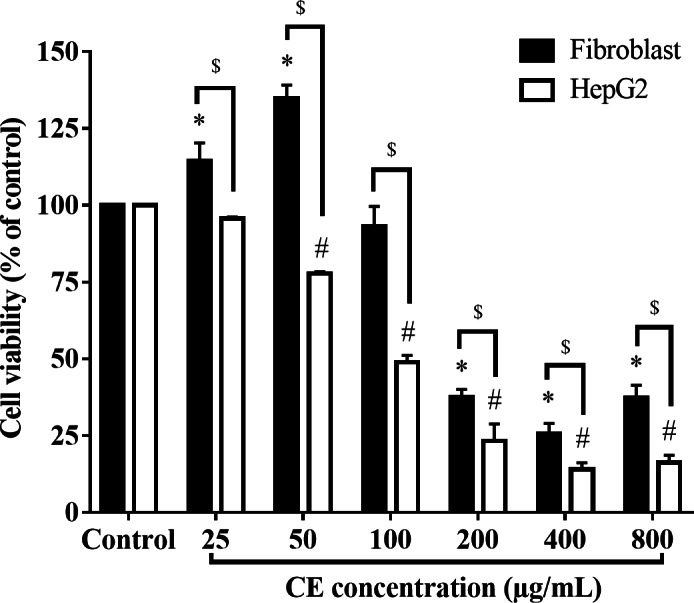
Effect of CE on HepG2 and HDFa cells viability. HepG2 cells (4 ×10^4^ cells/well) and HDFa (2 ×10^4^ cells/well) were treated with CE for 24 h. The percentage of viable cells was calculated against the control. The values were expressed as mean ±  SEM (*n* = 3). ^∗^*p* < 0.05 compared with HDFa control; ^#^*p* < 0.05 compared with HepG2 control; ^$^*p* < 0.05 HDFa compared with HepG2 at the same concentration by two-way ANOVA followed by SNK.

The cytotoxic effect of CE was markedly differently in cancerous versus the non-cancerous cells (Fig. 1). It was found that CE reduced the viability of HepG2 cells in a dose-dependent manner. At all treatment concentrations, CE exerted cytotoxic effects on HepG2 cells but showed significantly less effect on the cell viability of HDFa cells (*p* < 0.05). The viability of HepG2 cells considerably decreased at concentrations of CE ≥ 50 µg/ml, whilst that of HDFa cells began to decrease at CE ≥ 200 µg/ml. Interestingly, CE at low doses (25 and 50 µg/mL) was found to stimulate the growth of HDFa cells. After 24 h of treatment, the IC_50_ values of CE were 121.75 ±7.98 (µg/ml) and 301.16 ±27.71 for HepG2 and HDFa cells, respectively. Therefore, based on the IC_50_, 100 µg/ml of CE was chosen as the highest concentration of all subsequent experiments.

### Effect of CE on apoptosis

**Figure 2:**
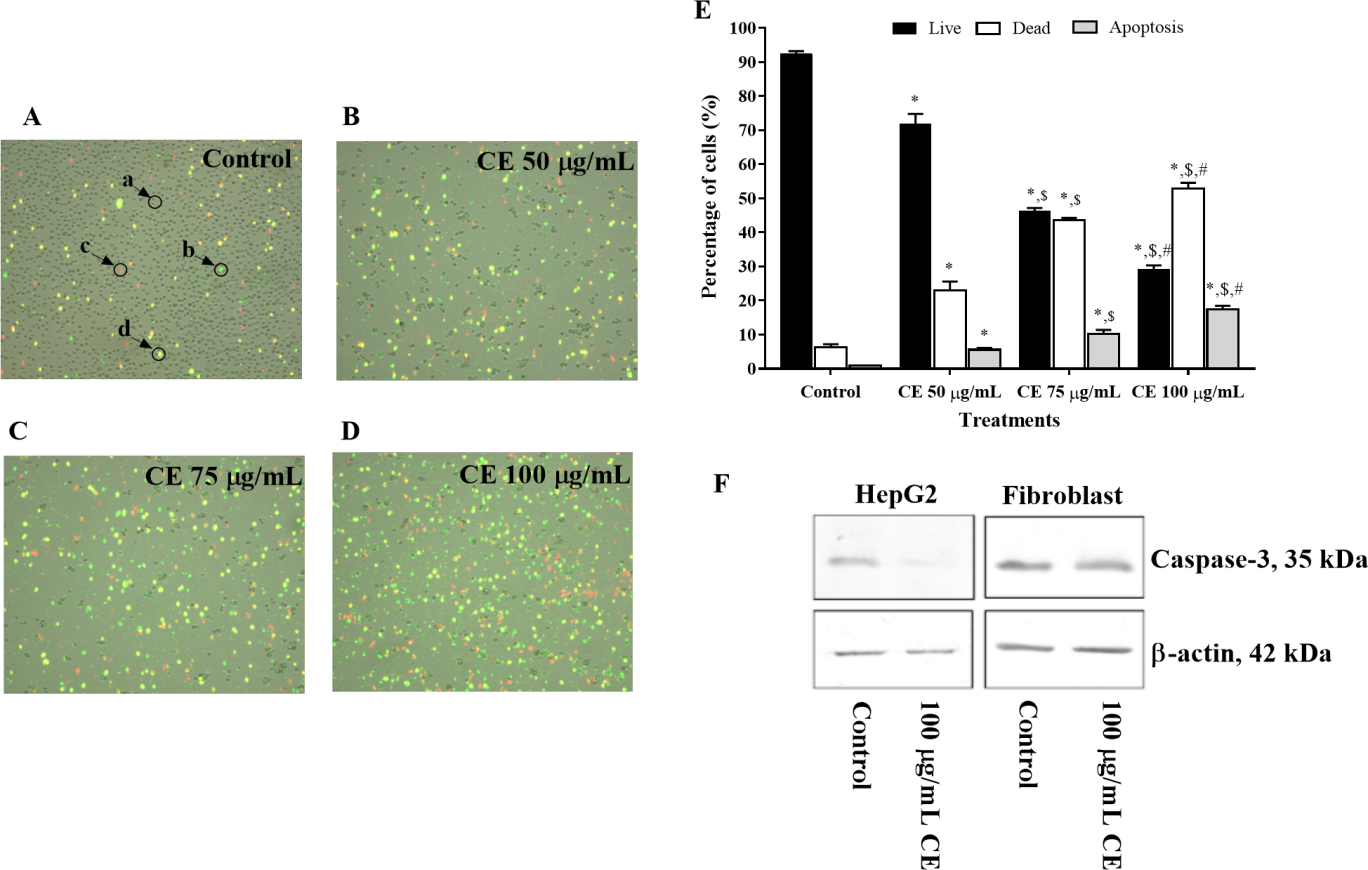
Effect of CE on apoptosis of HepG2 cells. (A–D) Annexin V and Propidium Iodide stained cells treated with various concentrations captured by Tali image-based cytometer (a, unstained cells designed as live cell; b, green cells are labeled with Annexin V and designed as apoptotic cell; c, d, red and yellow cells are labeled with PI or PI + Annexin V and designed as dead cells). (E) Percentage of live, dead, and apoptotic cells treated with CE for 24 h was determined by Tali™ apoptosis kit. The values were expressed as mean ±  SEM (*n* = 5). ^∗^*p* < 0.05 compared control; ^$^*p* < 0.05 compared with CE 50 µg/mL; ^#^*p* < 0.05 compared with CE 75 µg/mL by one-way ANOVA followed by SNK. (F) The expression of Procaspase-3 by Western blot.

Figs. 2A– 2D and 2E show the dose-dependent effect of CE-induced apoptosis in HepG2 cells. In this study, the apoptotic induction ability of CE was investigated using two dyes Annexin V Alexa Fluor^®^ 488 and propidium iodide. Under apoptotic conditions, phosphatidylserine is converted from the inner cell membrane to the outer surface of the cell membrane. Thus, the fluorescently labeled Annexin V (green color) detects apoptotic cells by binding to this exposed protein, meanwhile propidium iodide (red color) is used to determine the dead cells, demonstrated in Figs. 2A– 2D. Yellow colored cells indicate both Annexin V and propidium iodide staining, implying late apoptotic dead cells (Koç et al., 2018; Life Technology, 2013). The proportion of dead and apoptotic cells increased from the control group (CE 0 µg/ml) as the concentrations of CE increased from 50, 75 and 100 µg/ml (Fig. 2E).

In order to confirm that CE mechanistically induced apoptosis, the expression of executioner caspase-3 was investigated. It has been well established that cleavage of procaspase-3 plays a major “effector” role in apoptotic cell death (Walsh et al., 2008). This study showed that CE treatment (100 µg/mL) markedly decreased procaspase-3 protein expression Fig. 2F), indicative of caspase-3 cleavage (Boudreau, Peh & Hergenrother, 2019; Zaman, Wang & Gandhi, 2015). Taken together, these results suggest that CE dose-dependently induced apoptotic cell death in hepatocarcinoma cells.

### Morphological changes in HepG2 cells

**Figure 3:**
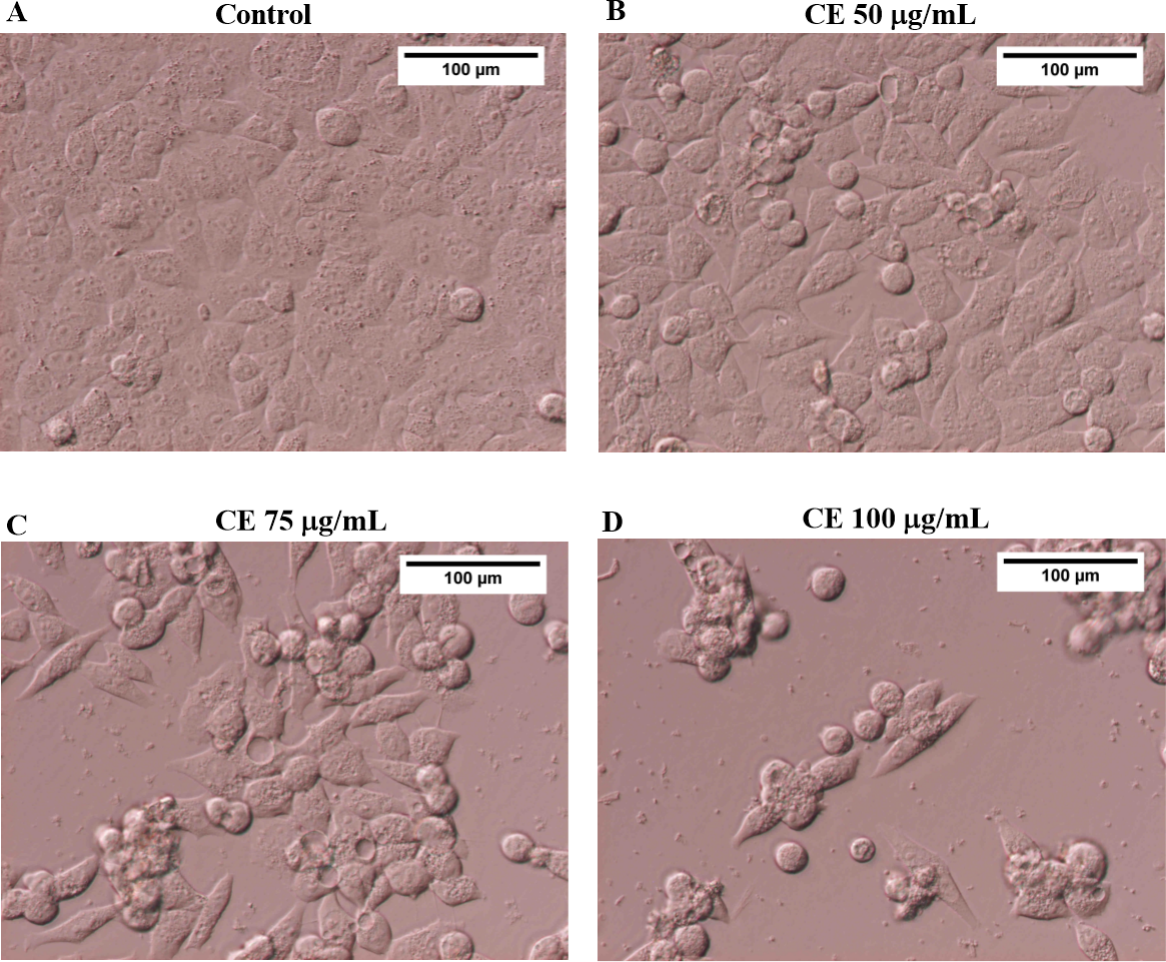
Morphological change of HepG2 cells after treatment with CE for 24 h (200X magnification). CE-treated HelpG2 cells morphology visualized under an inverted phase contrast microscope (A–D).

As shown in Fig. 3, the morphological changes of HepG2 cells were clearly shown via microscopy. In comparison to the healthy control, CE treated cells were rounded and shrunk while some cells were detached from the bottom of the wells. The changes in cell morphology were more noticeable when cells were treated with a higher dose of CE.

### Effect of CE on intracellular ROS and mitochondrial membrane potential

**Figure 4:**
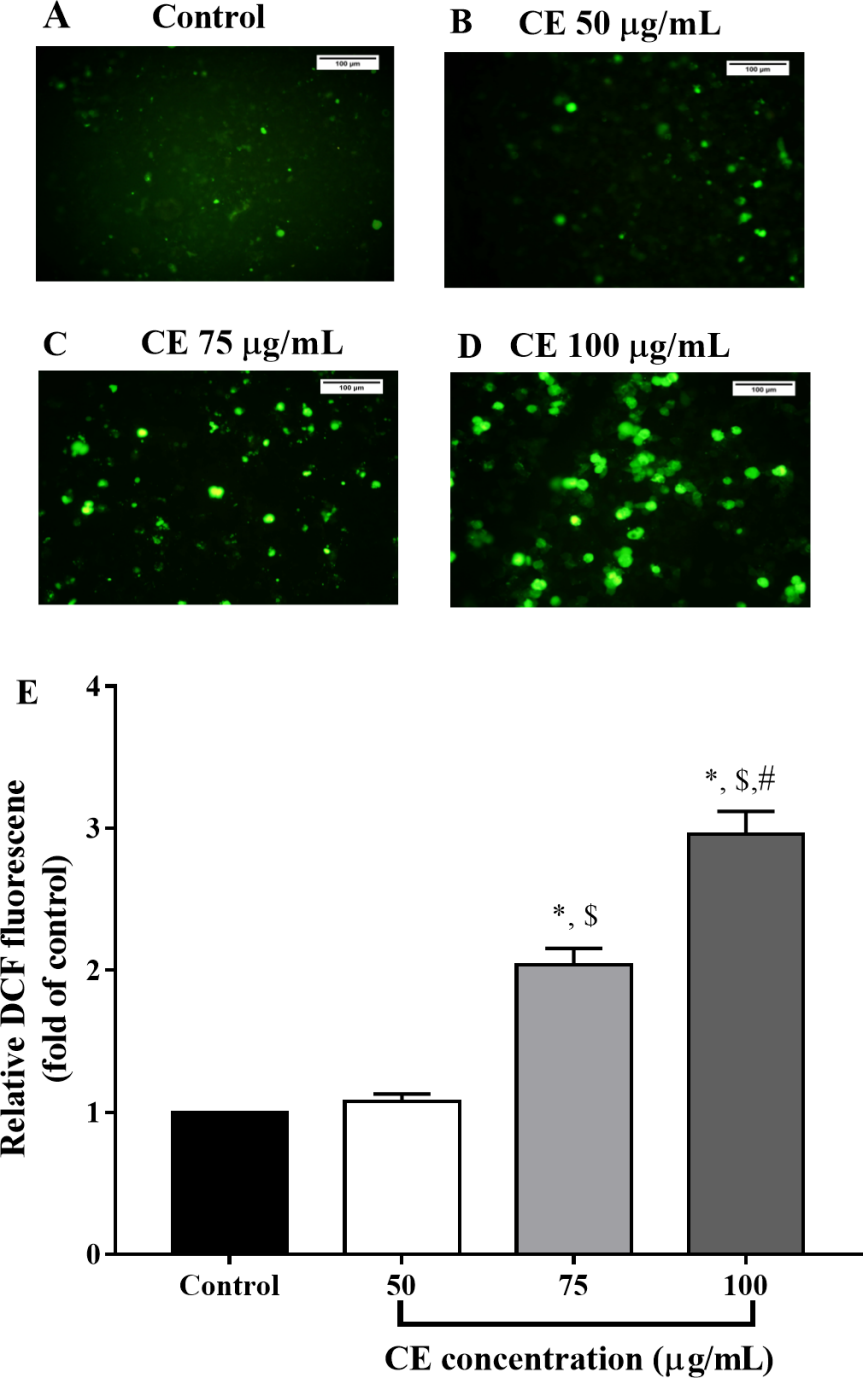
Effect of CE on intracellular ROS production. HepG2 cells were treated with CE for 12 h. The elevation of ROS was determined by using DCFH-DA dye. (A–D) ROS generated in cells was detected by phase-contrast fluorescence microscope (200X magnification; bar 100 µm). (E) The fluorescent intensity measured using fluorescent microplate reader. The values were expressed as mean ±   SEM (*n* = 3). ^∗^*p* < 0.05 compared control; ^$^*p* < 0.05 compared with CE 50 µg/mL; ^#^*p* < 0.05 compared with CE 75 µg/mL by one-way ANOVA followed by SNK.

**Figure 5:**
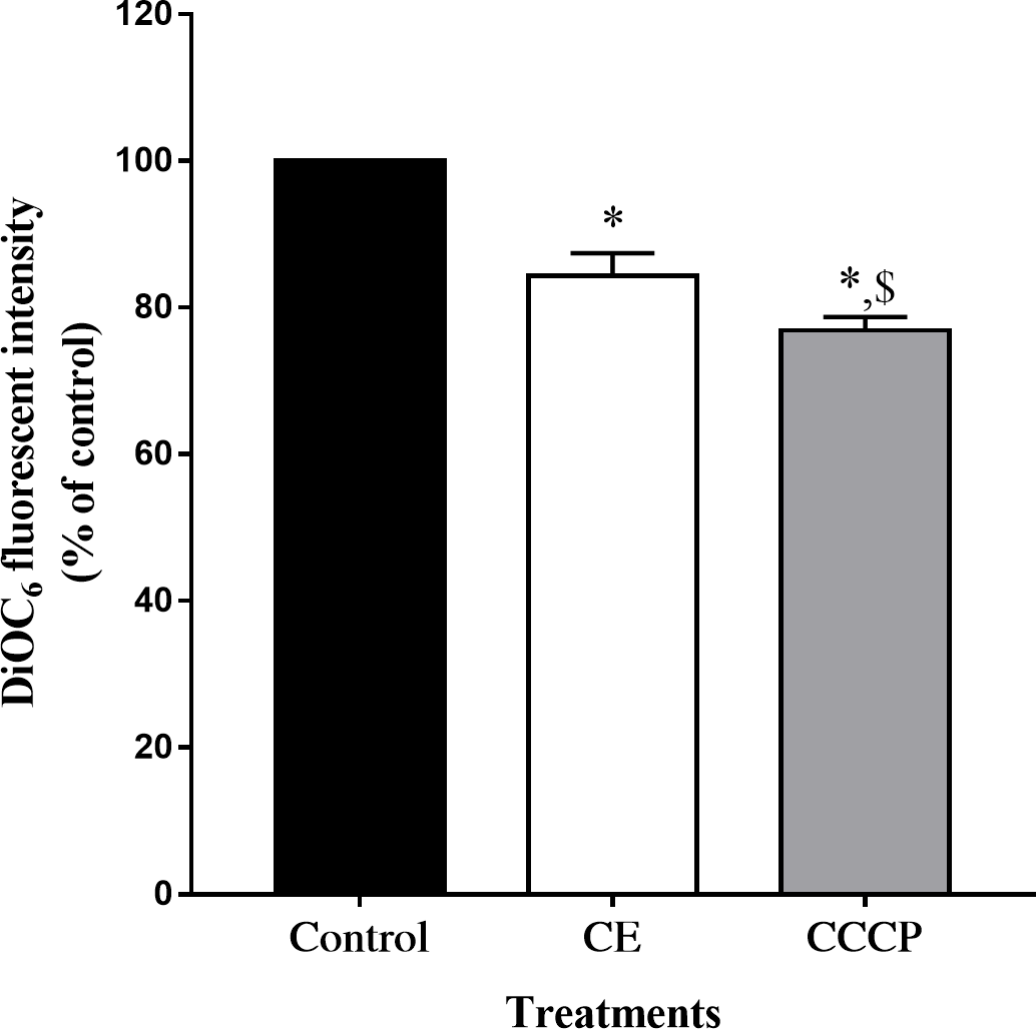
Effect of CE on mitochondrial membrane potential (Δ*ψ*_*m*_) of HepG2 cells. Cells were treated with CE 100 µg/mL for 24 h or 50 µM CCCP for 1 h and Δ*ψ*_*m*_ was measured using DiOC6 dye (Sigma, Missouri, USA). The values were expressed as mean ±  SEM (*n* = 3). ^∗^*p* < 0.05 compared control; ^$^*p* < 0.05 compared with CE by one-way ANOVA followed by SNK.

In comparison with the control, the intensity of green DCFH-DA fluorescence, indicative of intracellular ROS generation, significantly increased after CE treatment. After 12 h of treatment, it was found that intracellular ROS generated by CE at 75 and 100 µg/ml noticeably increased to approximately 2 to 3 folds with respect to the control (*p* <  0.05) (Fig. 4). As the accumulation of ROS has been associated to the disruption of Δ *ψ*_m_ (Huang et al., 2009; Marchi et al., 2012), we then examined the effect of CE on Δ *ψ*m using DiOC6 probe. Compared to the CCCP-mediated depolarization of Δ *ψ*_m_ (positive control), CE treatment (100 µg/ml) significantly decreased the Δ*ψ*m of HepG2 when compared to untreated cells (Fig. 5).

### Effects of CE on cell cycle distribution

**Figure 6:**
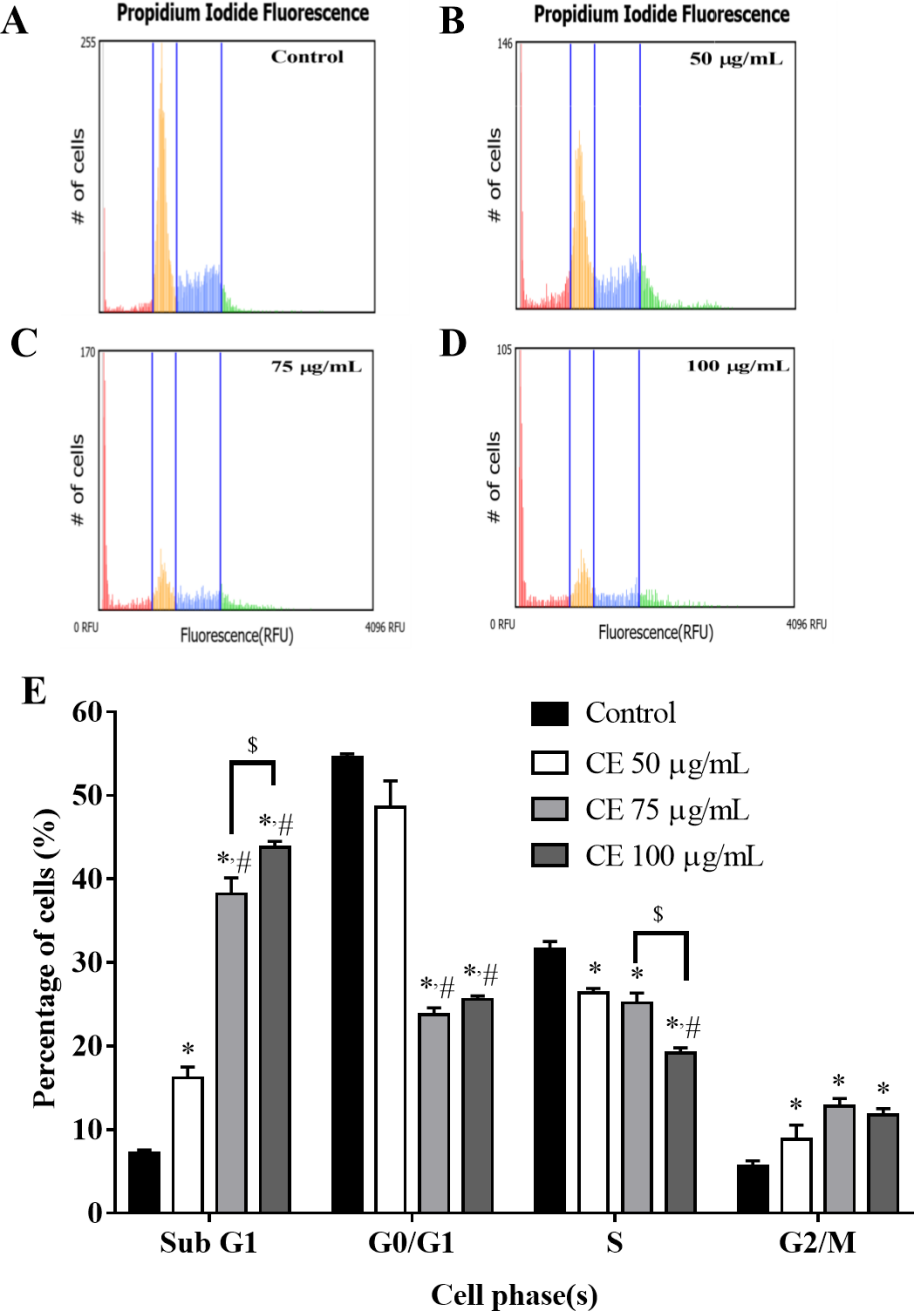
Effect of CE on cell-cycle distribution of HepG2 cells. HepG2 cells were treated with CE for 24 h. Cells were collected and stained by the Tali™ cell cycle kit. (A–D) Cell cycle stages of CE-treated cells with various concentrations as measured using a Tali image-based cytometer. Cells were classified into four distinctive phases: sub-G1 (red), G0/G1 (yellow), S (blue), and G2/M (green); (E) The percentages of HepG2 cells distribution in Sub-G1, G0/G1, S and G2/M phases. The values were expressed as mean ±  SEM (*n* = 5). ^∗^*p* < 0.05 compared with control; ^#^*p* < 0.05 compared with CE 50 µg/mL; ^$^*p* < 0.05 compared with CE 75 µg/mL by one-way ANOVA followed by SNK.

Cells treated with CE at different concentrations (50, 75, and 100 µg/ml) for 24 h showed an increase in sub-G1 phase and G2/M phase when compared with control (*p* <  0.05). CE treatment caused a majority of HepG2 cells to accumulate at sub-G1 phase. The proportion of cells in the sub-G1 phase of the control and the CE-treated group were 7.20 ± 0.37% and 43.80 ± 0.74%, respectively (Fig. 6).

## Discussion

In recent years, medicinal plants and active compounds from plants have become a reservoir of drugs discovery, particularly for anticancer drug researches (Qurishi et al., 2011). Anticancer agents from natural origins have been used clinically such as alkaloids from *Catharanthus roseus* or paclitaxel purified from *Taxus brevifolia* (Shukla & Mehta, 2015). In the present study, the anticancer activity of the aqueous extract from the stem bark of *C*. *cainito* in HepG2 cells was reported for the first time. Our results showed that the viability of cells was dramatically decreased at 50 µg/ml and more severely when the concentration of CE was increased. Human and rat fibroblast cells, including HDFa and NIH-3T3, have been widely used as a non-cancer cell comparison for studying cytotoxicity of agents and the underlying mechanisms involving cell cycle and apoptotic pathway (Chan et al., 2004; Felczykowska et al., 2015; Ocker et al., 2004). Interestingly, the CE showed less toxicity to fibroblast HDFa cells at any of the tested concentrations when comparing to HepG2 cells. For instance, HepG2 cells treated with CE 100 µg/ml induced approximately 50% cell death whereas there was no significant difference in the viability of HDFa cells when exposed to CE at the same concentration. This result indicated that CE was substantially more toxic to cancer cells than non-cancer cells.

Previous studies have demonstrated that cell apoptosis is associated with DNA fragmentation (Malgorzata et al., 2007; Riccardi & Nicoletti, 2006). Downstream regulation of this ROS-mediated mitochondrial pathway has been intensively studied and found that it is linked with cytochrome *c* release, caspase-3 cleavage, and p53 phosphorylation (Chen & Wong, 2008; Chen et al., 2008; Yang et al., 2013a). Apoptosis is programmed cell death which is disturbed in cancer cells. Hence, this programmed cell death was demonstrated to be an underlying mechanism of antitumor activity. The promotion of apoptosis in cancer cells is an important target in the development of anticancer drugs (Fulda, 2015; Pistritto et al., 2016). In this study, we found that CE increased apoptosis in HepG2 cells in a concentration-dependent manner (Figs. 2A– 2E). The apoptotic effects of CE was confirmed by the results from Western blot analysis. Fig. 2F shows that CE activated the cleavage of caspase-3, an effector caspase that has a central role in cell apoptosis. Our apoptotic result agrees with the other investigation that an ethyl acetate fraction of *C*. *cainito* fruit pulp possesses anticancer activity via inducing apoptotic and growth inhibition in U-2 osteosarcoma cells (Li et al., 2015). Furthermore, HepG2 cell morphology displayed characteristics of apoptotic cells (Fig. 3). Key characteristics of an apoptotic cell include cell shrinkage, membrane budding, extracellular exposure of phosphatidylserine, chromatin condensation and DNA fragmentation (Pistritto et al., 2016).

To explore the possible mechanism underlying CE-induced apoptosis in HepG2 cells, we measured intracellular ROS generation. Intracellular ROS level in HepG2 cells was significantly increased when treated with CE (Fig. 4). At the same time, HepG2 treated with CE also showed a decrease in mitochondrial membrane potential (Fig. 5). It was reported that ROS plays a major role in carcinogenesis. Nonetheless, a high level of ROS can suppress or kill cancer cells (Yang et al., 2013b). Excessive ROS can promote the apoptotic pathway by directly influencing mitochondrial functions and triggering the activation of caspase cascades (Simon, Haj-Yehia & Levi-Schaffer, 2000). Mitochondrial membrane permeabilization has been targeted in cancer treatment (Kroemer, Galluzzi & Brenner, 2007). The accumulation of ROS and the depolarization of Δ *ψ*m lead to the activation of the intrinsic apoptosis pathway in tumor cells (Huang et al., 2009). Thus, ROS and mitochondrial-dependent programmed cell death might be involved in the anticancer activity of CE in HepG2 cells.

Mitosis is the process of cell duplication in which one parent cell divides into two genetically identical daughter cells. During mitosis, chromosomes undergo duplication and segregation process which drive cell cycle in to 4 distinct phases. Those phases include: G0/G1 (rest/growth phase), S phase (synthesis), G2 phase, and M phase (mitosis). At every cell phase, there are checkpoints which control cell proliferation (Barnum & O’Connell, 2014) such as tumor suppressor proteins which induce cell cycle arrest and allow cells to attempt repair (López-Sáez et al., 1998). Therefore, new approaches for innovative cancer therapies is targeting cell cycle regulation (Diaz-Moralli et al., 2013). The result from cell cycle analysis revealed that CE dose-dependently increased the sub-G1 fractions and G2/M phase but decreased S and G0/G1 phases in HepG2 cell line (Fig. 6). It has been suggested that the increase in sub-G fraction indicate cell apoptosis (Halicka et al., 2002; Heishima et al., 2015). The upstream and downstream mechanisms underlying this effect, such as activation Cdc2, stimulation of Cdk inhibitors and G2/M arrest-related proteins, need further investigation.

Taken together, our findings suggest that the CE induces apoptosis in human hepatocarcinoma HepG2 cells via the ROS-mediated mitochondrial pathway, by increasing ROS production and inducing mitochondrial membrane depolarization. The ROS-mediated mitochondrial pathway has been widely proposed as a mechanism underlying anticancer properties of many plant products, for example, cajanol from *Pigeonpea* roots (Luo et al., 2010), delphinidin 3-sambubioside isolated from *Hibiscus sabdariffa* (Hou et al., 2005), and capsaicin, a pungent ingredient of red pepper (Huang et al., 2009).

Phytochemical constituents of *C*. *cainito* fruits have been reported to include alkaloids, flavonoids, phenols, steroids, saponins, tannins, and cardiac glycosides (Oranusi, Braide & Umeze, 2015). Nine flavonoids (quercetin, quercitrin, isoquercitrin, (+)-catechin, (-)-epicatechin, (+)-gallocatechin, (-)-epigallocatechin, gallic acid and myricitrin) have been indentified in *C*. *cainito* fruit (Luo, Basile & Kennelly, 2002). The *C*. *cainito* leaf extract has been proposed to contain antioxidative agents such flavonoids and triterpenoids (Sayed et al., 2019; Shailajan & Gurjar, 2014).

However, data of phytochemical constituents in bark stem is very limited. From our previous report, screening of phytochemicals of stem bark extract reveal that CE contained phenols, tannins, glycosides, terpenoids and saponin, but not flavonoids, steroids and alkaloids (Doan et al., 2018). In this study, the active ingredient responsible for the anticancer effects was not investigated. However, we believe that our present findings could provide some insights and spark further interest in deciphering the bioactive compounds in CE extract.

## Conclusions

In conclusion, the anticancer activity of *C*. *cainito* stem bark extract on human hepatocarcinoma HepG2 cells and its mechanisms of action are first reported herein. The present study shows that the extract significantly inhibits cell proliferation by an induction of apoptosis, which is evidently mediated by the generation of ROS, subsequently decreasing mitochondrial membrane potential and causing cell cycle arrest. The data suggest that CE exhibit anticancer properties and its therapeutic potential for the alternative management of human liver malignancy.

##  Supplemental Information

10.7717/peerj.10168/supp-1 Supplemental Information 1 Raw data from experiments

## Additional Information and Declarations

### Data Availability

The following information was supplied regarding data availability: The raw data are available in Data S1.
